# The housing first model (HFM) fidelity index: designing and testing a tool for measuring integrity of housing programs that serve active substance users

**DOI:** 10.1186/1747-597X-8-16

**Published:** 2013-05-03

**Authors:** Dennis P Watson, John Orwat, Dana E Wagner, Valery Shuman, Randi Tolliver

**Affiliations:** 1 Department of Health Policy and Management, Indiana University, Richard M. Fairbanks School of Public Health, Indiana University-Purdue University Indianapolis, 714 N. Senate Ave, Indianapolis, IN, 46202, USA; 2 School of Social Work, Loyola University Chicago, 820 N. Michigan Ave, Chicago, IL, 60611, USA; 3 Department of Psychology, Loyola University Chicago, 1032 W. Sheridan Rd, Chicago, IL, 60660, USA; 4 Midwest Harm Reduction Institute, Heartland Health Outreach Inc, 1207 W. Leland Ave, Chicago, IL, 6064, USA

**Keywords:** (3–10): Housing, Fidelity, Implementation, Instrument development, Mixed methods, Harm reduction, Low-demand

## Abstract

**Background:**

The Housing First Model (HFM) is an approach to serving formerly homeless individuals with dually diagnosed mental health and substance use disorders regardless of their choice to use substances or engage in other risky behaviors. The model has been widely diffused across the United States since 2000 as a result of positive findings related to consumer outcomes. However, a lack of clear fidelity guidelines has resulted in inconsistent implementation. The research team and their community partner collaborated to develop a HFM Fidelity Index. We describe the instrument development process and present results from its initial testing.

**Methods:**

The HFM Fidelity Index was developed in two stages: (1) a qualitative case study of four HFM organizations and (2) interviews with 14 HFM "users". Reliability and validity of the index were then tested through phone interviews with staff members of permanent housing programs. The final sample consisted of 51 programs (39 Housing First and 12 abstinence-based) across 35 states.

**Results:**

The results provided evidence for the overall reliability and validity of the index.

**Conclusions:**

The results demonstrate the index’s ability to discriminate between housing programs that employ different service approaches. Regarding practice, the index offers a guide for organizations seeking to implement the HFM.

## Background

Substance abuse is often offered as an explanation for the difficulty homeless individuals have accessing and maintaining housing. While substance abuse likely contributes to housing instability in some way (e.g., potential difficulty managing money and paying rent or increased potential for interpersonal disputes), it is also important to recognize that structural-level barriers to stable permanent housing also exist. Chief among these barriers are the stated and unstated policies determining who is eligible to access housing and the behaviors they must engage in to retain it [1]. Many policies guiding homeless services follow an abstinence-based approach requiring consumers to obtain sobriety (typically for 30–90 days) before they become eligible for housing. Likewise, individuals often must remain sober to keep their placement and/or advance to more independent levels of housing. This abstinence-based approach has been connected to problems such as higher consumer dissatisfaction and disengagement from services [2,3].

Developed in response to problems such as these, the Housing First Model (HFM) places lower demands on consumers. It has become the driving force of policies aimed at ending chronic homelessness due to its success engaging “hard-to-serve” individuals [4-6]. Despite this, wide diffusion coupled with a lack of clear implementation guidelines has led to confusion as to the elements of the model necessary for replication [7-9]. We created the HFM Fidelity Index in an effort to address the gap between HFM policy and community practice. In this paper we present results related to the development and testing of the index.

### Description of the HFM and its diffusion

The HFM was developed to serve individuals who are chronically homeless and who have been dually diagnosed with both a serious mental illness and a substance use disorder—a group that makes up anywhere from 10 to 20 percent of the total homeless population [4,5,10]. Pathways to Housing Inc. developed what is often credited as the first HFM program (then named the Consumer Preference Supported Housing Model) in the early 1990s. A key feature that distinguished the Pathways program from those following an abstinence-based approach was a lack of a sobriety requirement for admission to and retention of housing [11]. This lack of requirement is based in a harm reduction approach to services, which seeks to reduce the negative consequences related to substance abuse (and other high-risk behaviors) rather than eliminating substance use altogether [12,13].

In addition to reduced substance use and abuse [14], some of the key outcomes differentiating HFM from abstinence-based programs are: fewer emergency room visits and hospitalizations [15]; higher perceived choice in services [16,17]; reduced involvement in criminal activity [18]; and higher housing retention rates [19,20]. The United States Interagency Council on Homelessness and the National Alliance to End Homelessness became advocates of the HFM after programs based on its demonstrated ability to retain consumers in housing (i.e., residential stability) [4,6]. These endorsements resulted in rapid, nation-wide diffusion of the model over the past 12 years, which is largely related to local-level policies focused on ending chronic homelessness through a Housing First approach. This approach largely entails redirecting monies from traditional shelter services toward the development of more permanent and supportive housing options, particularly those that operate using the HFM [21].

Despite the intent of these policies, previous research has demonstrated challenges related to implementation of the HFM. In one study, George et al. [7] found that some providers who had experience working in an abstinence-based environment had difficulties understanding HFM policies and practices, particularly those related to harm reduction. Misunderstandings such as these can lead to modifications during the implementation process that can severely weaken a program model [22,23]. Indeed, Pathways staff have even begun to differentiate their program from other HFM programs as differences in implementation (and possible issues associated with it) have been recognized: “we [Pathways staff] refer to it as the Pathways Housing First (PHF) program to distinguish it from other programs that also identify with the Housing First approach” [24] (p. 4).

Seeking to better understand HFM implementation in the community, the U.S. Department of Housing and Urban Development (HUD) commissioned an exploratory study that sought to understand the characteristics of HFM programs [9]. The program characteristics the authors of this study point to include: (a) direct placement of consumers into permanent housing; (b) availability of supportive services without requirement to participate; (c) use of assertive outreach to engage reluctant consumers; (d) approaches to ensure relapse does not result in eviction; and (e) continuation of housing and case management services if clients leave for short time periods. This study was an important first step in understanding how the HFM has translated into community practice.

### Fidelity

Many factors can negatively affect an organization’s decision to adopt a specific evidence-based model for substance abuse intervention and its resulting implementation (e.g., size, resources, staff attitudes and education, local laws and policies) [25,26]. In this light, it is necessary for researchers, policy makers, and practitioners to have tools at their disposal for measuring the extent to which a model has been implemented in practice. This helps reduce the chance that outcomes, positive or negative, will be misappropriated to a model never fully implemented in practice, a phenomenon referred to as a Type III error in the implementation literature [27]. Fidelity measures are just such a tool, and research has established there is a positive association between fidelity and program outcomes [22,23]. Despite this, intervention effectiveness studies rarely pay attention to fidelity [28].

Fidelity has traditionally been conceptualized as strict adherence to the model as it was tested under scientific conditions [22]. However, a number of scholars have argued it is necessary for organizations to make adaptations to a model based on their particular circumstances [22,29,30]. For example, through a case study of a multisite fidelity assessment of individual projects providing substance abuse services, Orwin found that all 14 sites deviated to some degree from the implementation plan due to unique difficulties they each faced [31]. Similarly, Neumiller et al. found that challenges to implementing the Assertive Community Treatment (ACT) service model for people who were homeless with co-occurring disorders—a key ingredient of PHF programs [24]—resulted in modifications to the model in all 9 programs involved in their study, and that these modifications were made with knowledge that they would result in lower fidelity to the original model [32]. Matejkowski and Draine found that ACT services were often adapted among a sample of HFM programs due to the model’s focus on consumer choice in services [33]. For those who see adaptation as beneficial, program implementation is a complex process for which organizational context is an important factor, and modifications are allowable as long as the program delivers the “critical elements” that distinguish it from other models [22,34]. Identification of the critical elements provides a guide as to what can be modified during adaptation to local conditions [25,35].

While adaptations to the HFM might be appropriate and necessary in many instances, modifications that are in direct conflict with its basic underlying philosophy—those that are abstinence-based rather than harm reduction-based—are not appropriate. Therefore, development of a HFM fidelity instrument is critical to the successful implementation and measurement of the model. The focus of this paper is the development and testing of a HFM fidelity index.

## Methods

The study was carried out between August 2009 and August 2011. Researchers collaborated with staff at Heartland Health Outreach (referred to as Heartland hereafter). Heartland is a large social service organization that operates HFM programming, and also offers training and technical assistance to agencies seeking to implement HFM-based policies and practices. Researchers and Heartland staff worked in collaboration to ensure knowledge gained from the study would be appropriate for dissemination through both the academic and practice communities [36]. Procedures were approved by the Institutional Review Boards at Loyola University Chicago and Heartland Alliance.

### Development of the fidelity index

No fidelity instrument had been created at the time we started development of the HFM Fidelity Index. Recognizing wide diffusion of the HFM without fidelity guidelines had resulted in variations from the original model, we took a *bottom-up* approach to the development of the index that sought to identify and operationalize the *critical elements* of the HFM that differentiate it from the abstinence-based approach [37,38]. What this means is that we sought to understand the policies and practices of programs as they existed in a wide variety of contexts in an effort to identify those most central to the HFM’s success. We developed an initial index through two phases. We describe these phases and the initial instrument before discussing its final testing.

#### Phase 1

We provide a brief overview of Phase 1 in this section (methods and results for this phase are described in greater detail in another article [39]). Phase 1 was carried out by the first author, who employed a qualitative case study methodology with four local housing agencies [40]. Purposeful sampling techniques were used to select programs that were (a) strong examples of the HFM and (b) different enough in relation to program characteristics such as consumer capacity, population served, number of years operating a HFM, and housing type (single-site or multiple-site) to better assure similarities in themes would be related to the HFM rather than the organizational context [41]. Data were collected from 4 administrative interviews, 4 consumer focus groups (24 total participants), 3 staff focus groups (18 total participants), 21 consumer interviews, and 16 staff interviews. Staff received a $5 coffee shop card and consumers received a $30 grocery store card for their participation (consumers received a larger amount because staff completed the interviews during their work hours, and were therefore being compensated by their employers). Data collection and analysis were overlapping so incremental learning could guide collection efforts at subsequent levels [40]. Themes were identified both within and across cases as they related to the research questions. Emerging themes were discussed with administration at each agency and local housing experts, a qualitative approach to ensuring rigor and validity [41].

This process resulted in the identification of 6 broad elements of the HFM that were shared by the four organizations. (1) Each of the programs had *low-threshold admission policy (LTAP)* designed to place as few requirements as possible on potential consumers for program entry. Staff discussed the LTAP as the primary feature of their program that made it, and other Housing First programs, unique from the abstinence-based programs with which they were familiar. (2) *Harm reduction* was considered the practice or “tool” used to keep consumers housed. Harm reduction strategies do not require consumers to be abstinent from alcohol and drug use—harm reduction focused policies and practices stood out as the most critical element for running a successful Housing First program in all four sites. (3) *Eviction prevention* refers to a form of case management intervention aimed to prevent consumers from losing housing in light of lease violations. Because eviction of a consumer was an example of a programmatic failure in all of the organizations, eviction prevention was necessary for helping to assure program success. (4) *Reduced service participation requirements*, compared to those found in abstinence-based housing, were demonstrated to be important. Interview and focus group participants discussed how allowing consumer choice over their level of service participation was a powerful tool for facilitating positive change. (5) *Separation between property management and case management roles and responsibilities* was demonstrated to be important due to the effect on the consumer-staff relationship. While all programs had some separation between these two types of providers, it became increasingly difficult for consumers to develop trusting relationships with case managers as the lines between case management and property management roles blurred. (6) *Strategies to inform and educate consumers* about HFM policies and practices were important since their understandings of housing services were largely based on their histories with abstinence-based programs. Consumer-level data repeatedly demonstrated that education about the HFM was the mechanism that helped them attach meaning to the choices provided to them through the elements of harm reduction and reduced service requirements.

#### Phase 2

The goal of Phase 2 was to develop a more exhaustive list of elements with stronger operational definitions. To accomplish this goal, we carried out semi-structured phone interviews with “users” of the HFM (i.e., individuals who were Housing First program administrators or managers). The interview instrument for this stage was developed based on results from Phase 1 and a review of the existing HFM literature. Through this initial process, we developed a series of questions used to identify and create operational definitions for the elements included in the final index (a copy of this instrument can be obtained by contacting the first author). One hundred and twenty-nine of these questions asked users to determine how important they understood specific items to be to the HFM (on a scale from “0”/“not important” to “4”/“extremely important”). Additional questions inquired about such things as frequency, intensity, and duration related to specific items.

We recruited “users” from HFM programs located in the 25 largest urban areas in the country (where Housing First programs were likely to be) as identified by 2000 U.S. Census estimates. We identified 70 programs in these areas that operated using a HFM through an internet search and assistance from local government officials who oversaw the management of homeless service funds. Our goal was to recruit 20 users; however, we were only able to conduct 19 interviews due to time constraints. Five of these participants were removed from the sample after interviews were completed because we determined early in our analysis that they were employing a strict abstinence-based service approach that conflicted with the core philosophy of the HFM. Therefore, our final sample for Phase 2 comprised 14 HFM users from 12 cities (see Table 1). Participants received a $5 coffee shop gift card, and their program was entered into a drawing to win a $500 electronics store gift card.

**Table 1:** Location of phase 2 participants

**City**	**Number of programs selected**
1. Columbus	2
2. Dallas	1
3. Denver	1
4. Detroit	1
5. Houston	1
6. Indianapolis	1
7. Los Angeles	1
8. Minneapolis	2
9. New York	1
10. Philadelphia	1
11. San Francisco	1
12. Seattle	1
Total	14

In order to determine which items users understood to be most important to the HFM, we calculated descriptive statistics (mean and standard deviation) for each. We sorted all of the items by mean in ascending order so we could compare and discuss their relative importance (Table 2 displays the means and standard deviations for these items).

**Table 2:** Means and standard deviations for specific items inquired about in phase 2 “user” interviews

**Item**		**Mean**	**SD**
	Assertive community outreach directly to:		
1.	• Potential consumers	3.50	0.65
2.	• Hospitals	2.57	0.94
3.	• Shelters	3.64	0.63
4.	• Interim housing programs	2.07	1.07
5.	• Government agencies	2.43	0.76
6.	Specific staff dedicated to outreach	3.43	1.16
	Programs mainly target services towards:		
7.	• Adults	3.23	1.24
8.	• Single individuals not attached to a family unit	2.86	1.23
9.	• Those who are chronically homeless	3.79	0.60
10.	• Those who have serious and persistent mental illness	3.79	0.43
11.	• Those who have a substance abuse disorder	3.29	0.91
12.	• Those who are actively using substances	3.14	0.86
13.	• Those who demonstrate a desire to move towards abstinence	3.29	1.14
14.	• Those who do not have a prior felony conviction	1.79	1.25
15.	• Those who have good credit and/or no convictions	1.21	1.25
	New consumers are assessed for:		
16.	• Housing readiness	1.86	1.29
17.	• Substance use	2.71	1.07
18.	• Mental health status	2.93	0.92
19.	• Physical health	2.71	0.83
20.	• Financial stability	1.14	1.29
21.	• Benefits and entitlements	2.43	1.58
22.	• New consumers submit to urinalysis	0.36	0.84
	Consumers admitted on:		
23.	1. First come, first serve basis	1.92	1.19
24.	2. Assessed need/vulnerability	3.31	0.75
25.	Consumers to have benefits upon admission	0.93	1.21
26.	Consumers to have insurance upon admission (i.e., Medicaid, Medicare)	0.64	1.08
27.	Consumers to agree to money management as a precondition for admission	1.36	1.60
28.	Consumers agree to representative payee ship	1.14	1.29
29.	Educate incoming consumers on the principles of Housing First	3.14	0.66
30.	Housing available in multiple neighborhoods or community areas	3.14	1.10
31.	Allow consumers to change housing location once housed	2.00	1.18
32.	Temporary housing available to consumers while waiting for permanent placement	3.07	1.07
33.	Private landlords for housing sites	2.79	0.89
34.	Staff dedicated to locating housing stock	3.38	0.87
35.	Staff dedicated to building relationships with property managers	3.62	0.65
36.	Consumers do not share living spaces such as bedrooms, living rooms, bathrooms, or kitchens	2.71	1.44
	Housing property management to:		
37.	• Allow consumers to use alcohol in unit	2.85	1.52
38.	• Allow consumers to use alcohol away from property	3.57	0.76
39.	• Allow consumers to use illegal drugs in unit	1.85	1.77
40.	• Allow consumers to use illegal drugs away from property	2.92	1.55
41.	• Allow consumers to be intoxicated on housing property	3.00	1.04
42.	• Prohibit consumer use of any substances in unit	0.71	1.20
43.	• Prohibit consumer use of any substances at any time, in or away from unit	0.43	0.76
44.	Housing does not have time limits other than those defined by the standard lease/occupancy agreement	3.71	0.50
45.	Housing lease is not tied to any type of service agreements	2.86	1.23
46.	All consumers have representative payees	0.93	0.10
47.	Consumer has a representative payee when has trouble managing money	2.71	0.99
48.	Emergency funds available to assist consumers in need	3.14	0.86
49.	Consumer as the lease holder	2.79	1.22
50.	Agency as the lease holder	1.36	1.34
	Require regular housing inspection for:		
51.	• Cleanliness	2.93	0.92
52.	• Contraband	0.86	1.17
53.	Consumers participate in regular urinalysis to detect substance use	0.21	0.58
54.	Directly place consumers into permanent housing situation (rather than interim or safe haven)	3.07	1.14
55.	Allow consumer choice in housing location	2.93	0.92
56.	Keep active drug and alcohol users separately housing from non-users	1.21	1.12
57.	Consumers to be assigned case managers	4.00	0.00
58.	Consumers to have regular contact with a case manager as a condition of housing	1.93	1.54
59.	Case manager contact occurs in-person	3.50	0.67
60.	Reduce the number of face-to-face meeting with a case manager as a consumer demonstrates a growing level of stability	2.33	1.51
	Consumers to be able to define:		
61.	• Case manager meeting agenda	2.79	0.80
62.	• Case manager meeting time, within reason	2.71	0.61
63.	• Case manager meeting location, within reason	2.43	1.16
64.	Have an ACT Team	2.79	1.25
65.	Intensive case management services	3.00	0.96
66.	Consumer chooses level of engagement in services	3.21	0.70
67.	Supportive services located on housing site	2.23	1.36
68.	Consumers allowed to refuse supportive services	2.79	1.05
69.	Staff utilize assertive engagement with consumers to make services attractive	3.64	0.50
	Important that consumers with mental health issues:		
70.	• See a mental health practitioner	2.79	1.12
71.	• Are compliant with psychiatric medication	2.36	0.84
	Consumers with physical health issues:		
72.	• See a health care practitioner	3.07	0.62
73.	• Are compliant with their medication prescribed for physical health problems	2.57	0.94
74.	Consumer choose their own goals	3.79	0.43
75.	Low-demand approach to serving consumers	3.67	0.65
76.	Stage-based/stage-wise substance abuse treatment	2.38	0.96
77.	Harm Reduction approach to serving consumers	3.57	0.65
78.	Educate consumers about harm reduction	3.15	0.99
79.	Assess consumers for discharge readiness	3.08	0.95
	Terminate housing services based on:		
80.	• Excessive pedestrian traffic in and out of unit	1.21	0.80
81.	• Having people stay in unit who are not on the lease	2.00	1.41
82.	• Keeping the unit in an unclean, hazardous state	2.43	1.28
83.	• Excessive noise	1.79	1.18
84.	• Threats of violence	3.14	0.95
85.	• Physical violence	3.79	0.43
86.	• Relapse	0.14	0.36
87.	• Alcohol use in room	0.36	0.93
88.	• Illegal substances in room	1.50	1.65
89.	• Any illegal activity in the room besides use of illegal substances	2.31	1.44
90.	• Nonpayment of rent	2.29	1.33
91.	Have formal eviction prevention protocol	3.64	0.84
92.	Continue providing services if housing is lost	3.08	1.32
93.	Work with consumers to prevent homelessness in preparation for eviction from housing	3.86	0.36
94.	Work with consumers to locate new housing if evicted	3.57	0.65
95.	Have a staff member dedicated to eviction and/or homelessness prevention	2.07	1.90
96.	Eviction and/or homeless prevention specialist is full-time	2.30	1.70
97.	Follow-up with consumers after voluntary discharge from housing/services	2.71	1.14
98.	Hold housing for consumers if they leave for short periods	3.64	0.63
99.	Continue case management services while housing is being held	3.31	0.63
100.	Minimum education qualifications for case managers	3.00	0.88
101.	Have an ethnically and culturally diverse staff	3.86	0.36
102.	Have formal protocol for hiring ethnically and culturally diverse staff	3.07	1.07
	Have the following types of professionals at agency:		
103.	• Psychiatrist or psychiatric nurse practitioner	3.29	0.73
104.	• Licensed mental health professional (e.g., social worker, psychologists, therapist, or counselor)	3.00	1.04
105.	• Certified substance abuse counselor	2.36	1.22
106.	• Vocational rehabilitation specialist	2.36	0.84
107.	• Medical doctor, nurse practitioner, or physician’s assistant	2.57	1.16
108.	• Nurse	2.57	1.02
109.	• Peer counselors	2.86	1.23
110.	24/7 availability of at least one staff member	3.36	1.01
111.	Case managers are accessible (via phone) outside of normal working hours	2.07	1.27
112.	Case manager offices located in separate location from housing	1.54	1.61
113.	Separate program staff who work with property management to enforce rules and regulations of housing if case manager offices are onsite	3.00	1.00
114.	Allow staff to have flexible working schedules	2.93	0.83
115.	Staff meet regularly with a supervisor	3.64	0.50
	Important for staff to be trained in:		
116.	• Motivational interviewing	3.57	0.65
117.	• Crisis intervention	3.86	0.36
118.	• Harm reduction	3.64	0.63
119.	• 12-step model	1.93	0.10
120.	• Stages-of-change treatment	2.93	0.83
121.	• Cultural sensitivity	3.71	0.50
122.	• Other_____________	n/a	n/a
123.	• Interdisciplinary team meetings	3.57	0.51
124.	Program to engage in program evaluation or outcome measure activities	3.71	0.50
125.	Involve consumers in program decision making	3.21	0.80
	Agency to be involved in Housing First policy discussions at:		
126.	• Local level	3.77	0.44
127.	• State level	3.46	0.66
128.	• National level	3.15	1.14
129.	Utilize housing retention score as an indicator of successful Housing First programming	2.67	1.23

Through this process, we identified a total of 29 elements of the HFM, which we organized into 5 categories or overarching dimensions based on face validity. Our goal in this process was to create an instrument that would make conceptual sense to housing providers. Table 3 provides descriptions of each of the elements and their dimensions.

**Table 3:** **Dimensions and elements of the fidelity index and rational supporting inclusion in instrument**^**a**^

	**Description**
**Dimension I**	Human resources-structure and composition: Refers to the composition and structure of the staffing.
1. Diverse staff	Program staff highly reflects the diversity within the consumer population.
2. Minimum education requirements	At least 25% of case managers have a Master’s degree or higher.
3. Harm reduction and crisis intervention knowledge	Program provides or requires ongoing training in harm reduction and crisis intervention for staff [11].^b^
4. Staff availability	At least one staff member is available to consumers twenty-four hours a day, seven days a week [11].^b^
5. Clinical staffing	Program has psychiatric staff and mental health professional on staff or contract [11,17].
**Dimension II**	Program boundaries: Limits placed on who the program will serve and the responsibilities of key staff members.
6. Population served	Program serves only chronically homeless and dually-diagnosed individuals, and it houses current drug users [11].^b^
7 .Consumer outreach	There is a designated staff member dedicated to outreach or an outreach department [11].^b^
8. Case management responsibilities	Case management responsibilities are limited to case management.^b^
9. Termination guidelines	The program only terminates consumers who demonstrate violence, threats of violence, or excessive non-payment of rent.^b^
10. Termination policy enforcement	The service termination policy is consistently enforced.^b^
**Dimension III**	Flexible policies: Policies and rules are written to appropriately serve consumers with greatest need/vulnerability and to allow them maximum choice in terms of substance use and housing.
11. Flexible admissions policy	The program has formal protocol for admitting consumers with the greatest need/vulnerability [11].^b^
12. Flexible benefit/income policy	The possession of or eligibility for income benefits is not a prerequisite for housing.
13. Consumer choice in housing location	The program works with consumers to find desirable housing [11].^b^
14. Flexible housing relocation	The program always attempts to relocate consumers when they are dissatisfied with their current housing placement [11].^b^
15. Unit holding and continuation of case management	The program holds housing for hospitalization and incarceration for more than 30 days and program continues to offer case management services while unit is unoccupied [9].
16. Flexible with missed rent payments	The program is flexible with missed rent payments, but holds the consumer accountable.^b^
17. Flexible alcohol use policy	The program allows alcohol use and housing allows alcohol in units [9].^b^
18. Flexible drug use policy	The program allows illicit drug use and housing allows illicit drug use in units [9].^b^
19. Eviction prevention	The program has a formal policy and protocol to work with consumers to prevent eviction and has a staff member dedicated to eviction prevention.^b^
20. Consumer input into program	The program has formal and informal mechanisms for receiving and implementing consumer input.^b^
**Dimension IV**	Nature of social services: The structure, policies, and practices related to social services offered by the program. (there is some overlap with Dimension IV; however, this dimension refers specifically to social services)
21. Low-demand service approach	Consumers are not required to engage in any services except for case management in order to receive/continue receiving housing [9,11].^b^
22.	Harm reduction approach to service provision: Program uses a harm reduction approach and staff has a strong conceptual understanding [9,11].^b^
23. Regular in-person case management meetings	Consumers meet with their case managers 2-3 times a month on average, but program has a policy that more frequent meetings occur in the first 1-6 months after admissions [11].
24. Small case loads	Case managers have 10 or fewer consumers on their case load.
25. Ongoing consumer education	Consumers receive ongoing education in Housing First and harm reduction policies and practices.^b^
**Dimension V**	Nature of housing and housing services: The structure of housing and housing services offered by the program and/or private landlords.
26. Structure of housing	Housing is scattered-site in buildings operated by private landlords [11].^b^
27. Fast placement into permanent housing	The program places consumers into housing in one week or less [11].
28. Temporary housing placement	Temporary housing placement does not last more than one month.^b^
29. Consumer is lease holder for housing unit	100% of consumers are the lease holders of their unit [11].

^a^Citations reflect literature that guided rational for inclusion of element when Phase II findings were not conclusive and/or assisted in the final operationalization of the element.

^b^Phase I findings provided rational for inclusion when Phase II findings were not conclusive and/or assisted in the final operationalization of the element.

Next we developed an index that would capture variations in each of the 29 elements. We created five ordinal anchors for each of the 29 elements for which a “5” described the strongest level of implementation of the element and “1” described the weakest. We created operational definitions for each of the anchors using the descriptive statistics calculated from the 129 items in the user interview as a guide. Generally, those items with a mean of 3 or higher were incorporated into the anchor describing the strongest level of implementation, and items with smaller means were incorporated into those anchors describing weaker levels. There were times when the descriptive statistics did not provide enough information for the construction of categories or conflicted with our understanding of the model. In these instances, we consulted the Phase 1 findings and the Housing First literature to guide the decision making process (see Table 3).

The anchors for the elements were complex due to their qualitative nature. Therefore, we developed a series of interview questions to ensure the information necessary to identify each element’s correct anchor would be collected. To ensure face and content validity, researchers reviewed each of the elements, their dimensions, anchors, and interview questions with Heartland staff at multiple times throughout the instrument development process.

The instrument resulting from this two-phase process was designed to gather information about each of the 29 elements, background information about each program (e.g., type of housing offered, whether case managers were on-site or off-site, program self-identification as Housing First or abstinence-based, and how long the program had been in operation), and other data necessary to develop a housing retention score for each program (retention is the primary outcome of interest housing programs use to measure their success). Questions pertaining to each element were placed with its anchors in the survey. A copy of the final fidelity instrument is available from the first author.

### Testing of the instrument

#### Participating programs

HFM and abstinence-based programs were recruited for participation. Abstinence-based programs are an ideal comparison group for determining discriminant validity because the HFM was developed as a solution to the problems associated with this type of housing [11]. To be included in the sample all programs had to (a) primarily serve individuals, rather than families, and (b) provide permanent housing. We randomly selected 140 permanent housing programs from a publicly available list of all 2009 U.S. Department of Housing and Urban Development (HUD) grant award recipients [42]. Research assistants called organizations and asked administrative officials whether their program followed a Housing First or abstinence-based model, as well as requesting their organization to participate in the study. We acquired consent for participation from each program’s administrative office.

Our goal was to recruit a total of 40 HFM and 20 abstinence-based programs for participation in the study by randomly selecting programs from the list of 140 until our quota was met. We over-selected HFM programs in order to capture the diversity of programs that we understood to exist in practice based on previous research and the experience of Heartland staff. We completed 42 HFM and 13 abstinence-based interviews because of time restrictions related to our source of research funding. However, we removed one abstinence-based program and two HFM programs from the sample prior to analysis because we determined after the interviews that they did not meet the inclusion criteria despite the information administrators provided during the screening process (two did not primarily serve individuals and one did not provide permanent housing). We removed a fourth HFM program because significant parts of the interview were contradictory (the interviewee stated that staff were regularly trained in harm reduction, but also said the organization did not use harm reduction practices). The final sample consisted of 51 programs—39 Housing First and 12 abstinence-based—from 35 states.

#### Interview procedure

Program case managers (or staff members who provided case management services) were invited to participate in fidelity interviews. Case managers were chosen, rather than administrators or managers, because they are in a key “front-line” position to understand (a) the program’s policies and (b) the extent to which those policies are actually being implemented/practiced [43]. The interview took approximately 30–60 minutes to complete. Case managers received a $5 coffee shop gift card for their participation and their program was entered into a drawing to win one $500 or one of two $250 electronics store gift cards.

### Analysis and results

Subsequent to performing the fidelity interviews, we classified each program into one of three categories: (1) Abstinence-Based (AB; *n* = 12), (2) Housing First *with* abstinence-based principles and/or practices (HF/AB; *n* = 18), and (3) Housing First *without* abstinence-based principles and/or practices (HF; i.e., true Housing First programs; *n* = 21). Our reason for dividing the programs that self-designated as “Housing First” into two categories was that a large number of them were employing abstinence-based policies and practices that conflicted with the core philosophy of the HFM. In order to create the groups, we identified all questions in the survey that indicated the presence of abstinence-based policies and/or practices (e.g., Does the program explicitly refuse to admit active substance users?; Does policy dictate that the program terminate consumers for active substance use?; Does your program require drug and/or alcohol abstinence of all consumers? Does your program work with substance abusing consumers using an abstinence-based approach?) and we moved a HF program to the HF/AB category if answers to any of these questions indicated the presence of abstinence-based policies and/or practices. Table 4 displays descriptive statistics for each of the 29 elements by each housing type.

**Table 4:** Means and standard deviations for individual elements by housing type

	**AB (*n =*12)**		**HF/AB (*n =*18)**		**HF (*n =*21)**	
**Element**	**Mean**	**SD**	**Mean**	**SD**	**Mean**	**SD**
1. Diverse staff	3.42	1.31	3.89	1.02	3.76	1.26
2. Minimum education requirements	3.92	1.51	4.17	1.54	4.67	0.97
3. Harm reduction and crisis…	2.75	1.71	3.61	1.69	3.48	1.72
4. Staff availability	4.67	0.78	4.50	1.04	3.71	1.49
5. Clinical staffing	2.83	1.34	3.39	1.09	3.57	1.33
6. Population served	2.58	1.00	2.61	1.04	2.67	0.91
7. Consumer outreach	2.50	1.31	2.67	1.24	2.90	1.48
8. Case management responsibilities	1.92	0.67	2.28	0.83	2.86	0.96
9. Termination guidelines	1.25	0.62	2.44	1.42	2.90	1.14
10. Termination policy enforcement	4.00	1.13	3.83	1.38	3.38	1.28
11. Flexible admissions policy	3.25	1.36	3.50	1.34	3.52	1.25
12. Flexible benefit/income policy	3.50	1.17	4.50	0.71	4.00	1.18
13. Consumer choice in housing…	2.67	1.78	1.83	1.15	3.43	1.43
14. Flexible housing relocation	2.08	1.44	1.61	1.04	3.14	1.53
15. Unit holding and continuation…	4.08	1.31	3.50	1.65	4.14	1.28
16. Flexible with missed rent payments	3.25	1.42	3.00	1.33	3.81	1.33
17. Flexible alcohol use policy	1.67	1.37	3.61	1.75	4.62	0.80
18. Flexible drug use policy	1.08	0.29	1.83	1.34	3.10	1.04
19. Eviction prevention	3.00	1.04	2.83	0.79	3.52	0.87
20. Consumer input into program	4.08	1.24	4.17	1.10	4.14	1.06
21. Low-demand service approach	3.50	1.45	3.72	1.23	4.10	1.04
22. Harm reduction approach…	2.08	1.16	3.00	1.41	4.76	0.54
23. Regular case management…	2.42	1.73	2.06	1.66	2.62	1.75
24. Small case load	3.83	0.94	3.78	1.17	4.10	0.94
25. Ongoing consumer education	1.00	0.00	2.17	1.29	2.81	1.29
26. Structure of housing	3.42	1.51	2.72	1.71	4.48	1.21
27. Fast placement into…	2.83	1.53	3.33	1.78	2.57	1.54
28. Temporary housing placement	2.67	1.87	3.00	1.97	1.71	1.38
29. Consumer is lease holder…	3.67	1.97	4.83	0.51	3.57	1.89

Next, we summed the scores for each of the 29 elements to create an *overall fidelity score*. A program could score anywhere from 29 (lowest fidelity/least ideal implementation of the elements) to 145 (highest fidelity/most ideal implementation of the elements).

We developed a number of questions to test the reliability and validity of the instrument. Each of these questions and the analysis approach we used to test them are listed below. All analyses were performed using SAS 9.3 statistical software. Because of the multiple steps involved in testing the instrument*, we have combined a description of each analysis, its results, and some discussion in the sections that follow* for the purpose of of providing greater clarity to the reader*.*

### Question 1: Do all 29 elements contribute to the reliability of the instrument

To investigate the relationship between scores on individual elements and the total fidelity score, we calculated item-total correlations, which assist in determining which elements contribute to the index’s reliability more than others. We expected that there would be variation in the relationship between each element and overall fidelity. We further expected that removing the negatively correlated items from the instrument would improve its internal consistency (i.e., raise Cronbach’s alpha).

Table 5 displays correlation coefficients demonstrating the relationships between the overall fidelity score and each of the elements. The first column displays correlation coefficients related to all 29 of the original elements. A correlation of 0.30 or higher is considered desirable when interpreting item-total correlations [44]. However, we chose to only eliminate elements with negative correlations because we consider the face validity established using the methods in the previous two phases to be a strength of the instrument.

**Table 5:** Item-total correlations for individual elements

	**All programs (*n*= 51)**	
**Element**	**Total fidelity score = 145**	**Total fidelity score = 120**
1. Diverse staff	-.023	---
2. Minimum education requirements	0.19	0.14
3. Harm reduction and crisis…	0.32	0.26
4. Staff availability	-.01	---
5. Clinical staffing	0.33	0.33
6. Population served	0.27	0.27
7. Consumer outreach	0.15	0.16
8. Case management responsibilities	-.030	---
9. Termination guidelines	0.33	0.26
10. Termination policy enforcement	-.022	---
11. Flexible admissions policy	0.01	0.01
12. Flexible benefit/income policy	0.10	0.10
13. Consumer choice in housing…	0.35	0.45
14. Flexible housing relocation	0.45	0.55
15. Unit holding and continuation…	0.30	0.31
16. Flexible with missed rent payments	0.31	0.35
17. Flexible alcohol use policy	0.57	0.56
18. Flexible drug use policy	0.50	0.54
19. Eviction prevention	0.22	0.27
20. Consumer input into program	0.33	0.31
21. Low-demand service approach	0.24	0.19
22. Harm reduction approach…	0.50	0.51
23. Regular case management…	0.03	0.08
24. Small case load	0.05	0.06
25. Ongoing consumer education	0.55	0.59
26. Structure of housing	0.41	0.50
27. Fast placement into…	0.06	0.08
28. Temporary housing placement	0.08	0.09
29. Consumer is lease holder…	-.25	---

The first column in Table 5 displays correlation coefficients related to all 29 of the original elements, 5 of which had negative relationships with the overall fidelity score. The second column in the table displays scores after removing the negatively correlated items and adjusting the total possible fidelity score to 120.

Table 6 displays *Cronbach’s alpha* coefficients for the overall index and each of the subdimensions before and after the removal of the negatively correlated ingredients. A score of 0.70 is the minimum desired for establishing internal consistency of an instrument [45]. As the table demonstrates, removal of the 5 negatively correlated items improved the internal consistency of the overall index. Only one of the subdimensions, flexible policies, was close to attaining a minimally desirable alpha coefficient in both analyses. This likely represents poor grouping of the elements given the internal consistency of the overall scale.

**Table 6:** Reliability of housing first dimensions before and after elimination of items with negative item-total correlations

**Dimension**	**Cronbach’s alpha**	**Cronbach’s alpha**
	***(with original 29 items)***	***(with final 24 items)***
I. Human resources (structure and composition)	0.39	0.39
II. Program boundaries	0.17	0.05
III. Flexible policies	0.66	0.67
IV. Nature of social services	0.35	0.35
V. Nature of housing and housing services	0.26	0.38
Overall	0.68	0.75

**Figure 1:**
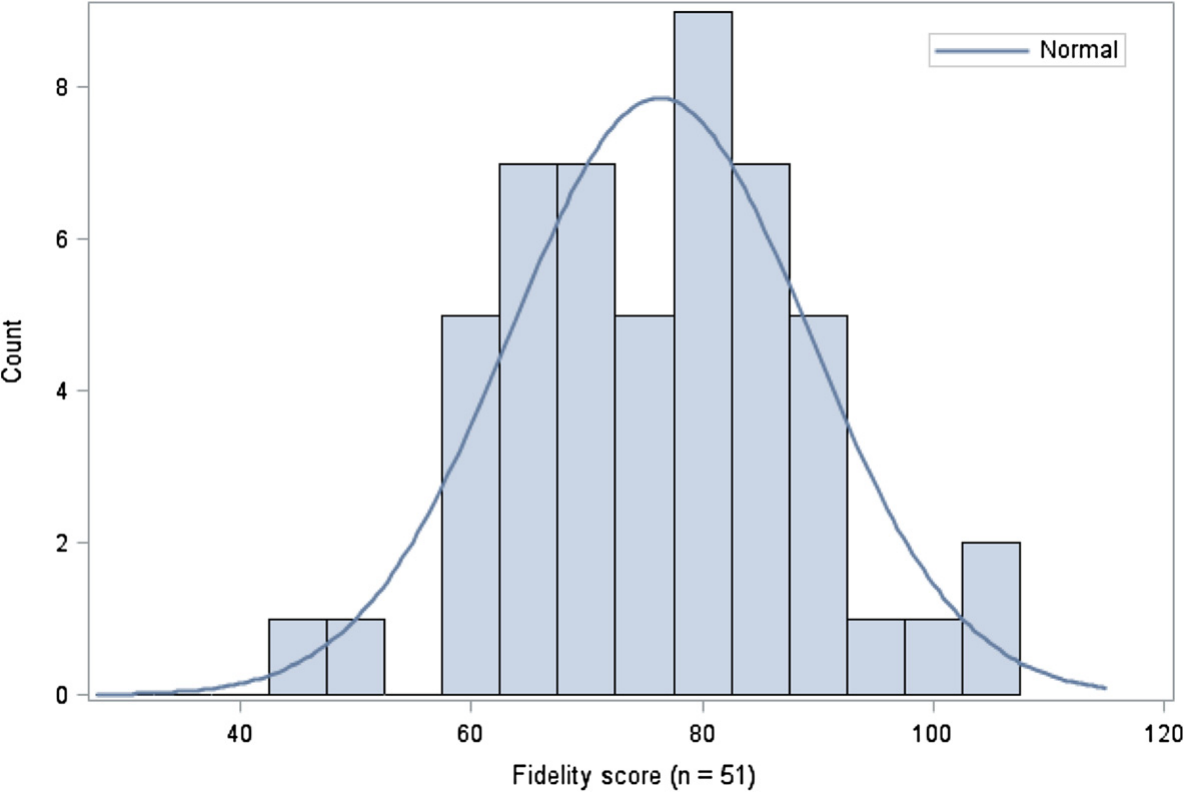
Histogram of adjusted fidelity scores (total possible score = 120).

Subsequent analyses were conducted using items from the adjusted instrument. Figure 1 displays a histogram of the adjusted fidelity scores.

### Question 2: Are there differences in mean fidelity scores between groups (i.e., Program Type)

Finding differences in mean fidelity scores between the three groups (AB, HF/AB, HF) is useful because it confirms that we formed the groups correctly and assists in establishing *discriminant validity* (i.e., fidelity scores should be higher among HF programs). We conducted a trend analysis in ANOVA with program type as the independent variable and fidelity score as the dependent variable [44]. We predicted that there would be a linear pattern where HF/AB programs would have higher mean fidelity than AB programs and HF programs would have a higher mean fidelity than HF/AB programs.

**Figure 2:**
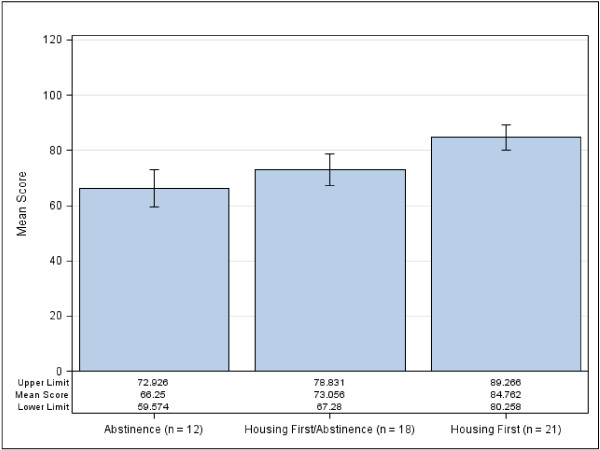
Mean fidelity scores with confidence limits by program type.

The mean fidelity score for the entire sample was 76.27 (SD=12.94). Figure 2 demonstrates the mean fidelity score for each of the program types with confidence limits. Results demonstrate that fidelity scores between the three groups were significantly different from each other in the predicted pattern, *ψ*_3_ = 18.51, *t*(48) = 4.79, *p* < .0001. HF programs had the highest mean fidelity score at 84.76 (*SD* = 9.89; *n* = 21; 95% CI [59.57, 72.93]), followed by HF/AB at 73.06 (*SD* = 11.61; *n* = 18; 95% CI [67.28, 78.83]), and AB programs at 66.25 (*SD* = 10.51; *n* = 12; 95% CI [80.26, 89.27]).

Thus, we found a key strength of the instrument is that it is able to differentiate between program types. This ability is rooted in the intensive qualitative work of the previous phases. It is through this work we were able to identify incompatibilities between the integration of abstinence-based policies and procedures and the underlying philosophy of the HFM. This provided the conceptual logic upon which we were able to recognize and create operational definitions for two different types of HFM programs (HF and HF/AB), which may be useful for those seeking to implement and or measure the HFM at varying levels. This is particularly true for housing agencies that seek to develop HFM implementation plans while facing structural, policy, and/or philosophical barriers that might limit their ability to carry out harm reduction activities.

The relatively small mean difference between AB and HF (18.51 points on a scale of 120) is notable. This is likely due to the fact that a number of the elements included in the instrument are aspects of quality housing programming that are not limited to the HFM. Future research warrants the investigation and possible removal of such elements from the index and/or weighting of those elements demonstrated to be more central to the model.

### Question 3: Is fidelity score related to housing retention

In order to determine *criterion validity*, we calculated *Spearman’s rho correlations* between total fidelity score and two measures of housing retention employed by Heartland as part of its federal funding requirements. We calculated the first measure of housing retention (HR1) by dividing the total number of consumers who had been served by the program for a minimum of 12 months (including those who were served 12 months before entering the fiscal year and those who were served 12 months during the fiscal year regardless of whether they left the program during the fiscal year or not) by the total number of consumers served that fiscal year. We calculated the second measure (HR2) by dividing the total number of consumers who remained in the project from the first to the last day of the fiscal year (i.e., those who did not exit the project regardless of their length of stay) by the total number of consumers served during the year. We expected that fidelity would be positively associated with both types of housing retention.

**Figure 3:**
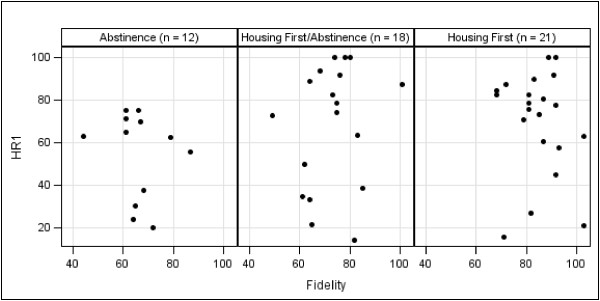
Scatterplot of First Housing Retention Score (HR1) and fidelity score by program type.

**Figure 4:**
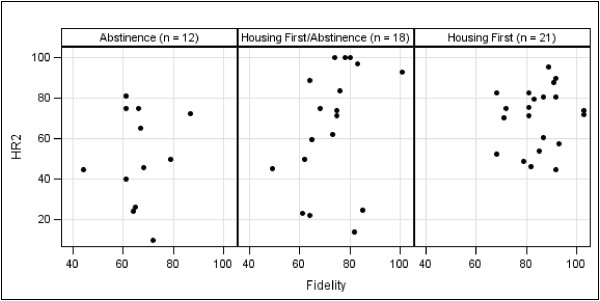
Scatterplot of Second Housing Retention Score (HR2) and fidelity score by program type.

Scatterplots of housing retention scores and fidelity are depicted in Figures 3 and 4. We calculated Spearman’s rho correlations between fidelity scores and both measures of housing retention. HR1 was not significantly associated with fidelity (*r*_*s*_ = .14, *p* = .32), and HR2 had a significant but weak correlation (*r*_*s*_ = .33, *p* < .05).

There are two possible explanations for the lack of correlation between fidelity and HR1. As discussed above, programs operating using abstinence-based policies and procedures often have difficulty retaining hard-to-serve consumers. Therefore, the HR1 score might be inflated in AB and HF/AB programs due to the increased emphasis this measure places on consumers who are more stable (i.e., those who have been housed for 12 months or more). A second possible explanation is AB and HF/AB programs are more likely to admit consumers they understand to be more stable and who are likely to be successful in their programs, a process known as “creaming” [43]. This is particularly true regarding the AB programs that only accept consumers who have demonstrated the ability to remain abstinent for some time period. Contrasting this, HF programs do not have prerequisites for consumer admission. Because it results in differences in consumer populations across program types, creaming could lead one to conclude that the program, as opposed to traits of the consumer, is responsible for positive consumer outcomes.

In contrast, it is likely that we found a significant relationship between fidelity and HR2 because this puts less weight on consumer stability. That is, the measure takes into account that HF (and to some extent HF/AB) programs are more likely to take in higher risk individuals due to the presence of a low-threshold admission policy. Indeed, it can be argued that the HR2 measure is more appropriate for understanding the relationship between fidelity and outcomes for this reason.

## Conclusions

The development of the HFM Fidelity Index is an important step in ensuring the quality and consistency of HFM implementation. Our results demonstrate the index was generally valid and reliable. Arguably the most successful result is its ability to discriminate between the three different types of housing described. Overall, the results point to those elements related to harm reduction as being the most critical to the HFM. Despite this, a number of Housing First programs participating in the study had abstinence-based approaches to service provision that stood in direct conflict to a harm reduction approach. This confirms results from previous research that demonstrate how misunderstandings of the HFM result in programs having flexible admissions policies coupled with strict abstinence-based rules by which consumers must abide in order to keep their housing [7]. This is problematic considering the HFM was developed due to the difficulty “hard-to-serve” consumers have remaining permanently housed in programs with rules such as these [11].

Despite promising results, there are study limitations that need to be considered. Resource and time constraints prevented us from collecting data that would allow for the measurement of reliability beyond internal consistency (e.g., inter-rater, test-retest). Second, the Cronbach’s alpha coefficient demonstrates that the index is reliable overall; however, there is need to further investigate the organization of the subdimensions based on the results. Third, our sample was relatively large for an organizational-level study; however, future studies with larger sample sizes would allow for additional analyses. Fourth, the findings might have been stronger had we eliminated elements with low item-total correlations from the instrument. We chose not to eliminate these elements because we see this study as a first step in the development of the instrument. Future work might justify their removal. Finally, the possibility of creaming (i.e., the tendency to recruit or admit only those consumers who are likely to be successful) within programs might be a confounding factor related to the housing retention measures. The inability to control for this issue demonstrates a limitation of the community-based research design; however, this is a reality of conducting research in naturalistic settings.

Despite the stated limitations, the HFM Fidelity Index does hold potential for researchers and practitioners. While we recognize one hundred percent fidelity is neither a goal nor a possibility for many organizations, the index offers a guide they can use to make implementation decisions and assess the quality of programming during the sustainability phase that follows implementation based on the level of fidelity they decide appropriate. The need for implementation guidelines has become even more important as the HFM is expanding beyond the United States [46]. Since we began our study, Pathways to Housing has also published an Essential Ingredients Checklist that should prove useful as an implementation guide [24]. However, Pathways has stated the checklist was designed to measure fidelity to the PHF model, and might not be appropriate for measuring fidelity in programs that have made adaptations, whether purposeful or accidental, to their model. Those programs specifically seeking to implement the PHF model should consult the Pathways fidelity checklist for guidance. Therefore, our index is likely to have greater flexibility when it comes to measuring the wide range of HFM programs that exist in practice. Finally, from a policy perspective, the index can be used by funders that support the HFM to help them assure their monies are being used in an appropriate fashion by programs that represent themselves as “Housing First.”

Regarding next steps, we would like to investigate other approaches to establishing reliability of the index and its subdimensions. One of our primary goals is to collect a larger sample that will allow us to test the index using Item Response Theory and to compare those results to those obtained using classical test theory methods. This will assist in identifying those elements that are not truly representative of the underlying construct (i.e., the HFM), thus improving the instrument's validity [47]. We also plan to explore different approaches for identifying and organizing the subdimensions beyond face validity, which will potentially lead to stronger internal consistency.

## Abbreviations

AB: Abstinence-based programs that were part of the study sample; HF: Housing First programs without abstinence-based principles that were part of the study sample; HF/AB: Housing First programs with abstinence-based principles that were part of the study sample; HFM: Housing First Model; HR1: A measure of housing retention calculated by dividing the total number of consumers who remained in the program for at least 12 months by the total number of consumers served during the contract year; HR2: A measure of housing retention calculated by dividing the total number of consumers who remained in the project during the contract year by the total number of consumers served during the contract year; HUD: U.S. Department of Housing and Urban Development; LTAP: Low-threshold admission policy; PHF: Pathways Housing First.

## Competing interests

The authors have no competing interests to declare.

## Author’s information

DPW is Assistant Professor in the Richard M. Fairbanks School of Public Health and Faculty Research Associate at the Center for Health Policy at Indiana University-Purdue University Indianapolis.

JO is Assistant Professor in the School of Social Work at Loyola University Chicago.

DW is Doctoral Candidate in the Department of Psychology at Loyola University Chicago.

VS, is the Associate Director of Heartland’s Midwest Harm Reduction Institute in Chicago.

RT is Director of the Illinois Co-Occurring Center for Excellence at Heartland Health Outreach, Inc.

## Authors’ contributions

DPW was the primary investigator for the entire study participated in the drafting of the manuscript. JO participated in the study design and the drafting of the manuscript. DW was project manager and statistical analyst for the project and participated in the drafting of the manuscript. VS participated in the research design and analysis. RT provided significant input regarding the interpretation of the data as it related to practice. All authors read and approved the final manuscript.
